# Intermittent mechanical loading on mouse tibia accelerates longitudinal bone growth by inducing PTHrP expression in the female tibial growth plate

**DOI:** 10.14814/phy2.16168

**Published:** 2024-08-01

**Authors:** Sarah McGarry, Karen Kover, Daniel P. Heruth, Mark Dallas, Xinxin Jin, Shufang Wu, Francesco De Luca

**Affiliations:** ^1^ Division of Endocrinology Children's Mercy Hospitals Kansas City Missouri USA; ^2^ Department of Pediatrics University of Missouri‐Kansas City‐School of Medicine Kansas City Missouri USA; ^3^ University of Missouri‐Kansas City‐School of Dentistry Kansas City Missouri USA; ^4^ Department of Physiology and Pathophysiology, School of Basic Medical Sciences Xi'an Jiaotong University Xi'an Shaanxi People's Republic of China; ^5^ Center for Translational Medicine The First Affiliated Hospital of Xi'an Jiaotong University Xi'an Shaanxi People's Republic of China

**Keywords:** growth plate, longitudinal bone growth, mechanical loading

## Abstract

It is not clear as to whether weight bearing and ambulation may affect bone growth. Our goal was to study the role of mechanical loading (one of the components of ambulation) on endochondral ossification and longitudinal bone growth. Thus, we applied cyclical, biologically relevant strains for a prolonged time period (4 weeks) to one tibia of juvenile mice, while using the contralateral one as an internal control. By the end of the 4‐week loading period, the mean tibial growth of the loaded tibiae was significantly greater than that of the unloaded tibiae. The mean height and the mean area of the loaded tibial growth plates were greater than those of the unloaded tibiae. In addition, in female mice we found a greater expression of PTHrP in the loaded tibial growth plates than in the unloaded ones. Lastly, microCT analysis revealed no difference between loaded and unloaded tibiae with respect to the fraction of bone volume relative to the total volume of the region of interest or the tibial trabecular bone volume. Thus, our findings suggest that intermittent compressive forces applied on tibiae at mild–moderate strain magnitude induce a significant and persistent longitudinal bone growth. PTHrP expressed in the growth plate appears to be one growth factor responsible for stimulating endochondral ossification and bone growth in female mice.

## Graphical abstract

Cyclical mechanical strain was applied three times a week for 4 weeks to the right tibia of seventy‐five 4‐week‐old mice. By the end of the 4‐week loading period, the longitudinal growth of the loaded tibiae was significantly greater than that of the unloaded left tibiae. In addition, we found a greater expression of PTHrP in the loaded tibial growth plates of the female mice. Intermittent compressive forces applied on mouse tibiae induce a significant longitudinal bone growth and increased PTHrP expression in the female tibial growth plate.

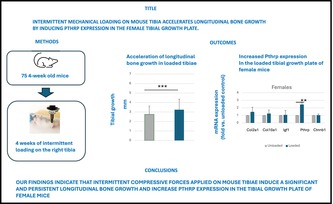

## 1 INTRODUCTION

Mechanical stimuli resulting from weight loading play a critical role in bone modeling and remodeling in mammals. On the other hand, several reports have shown inconsistent effects of weight bearing on longitudinal bone growth (Reich et al., 2005): some studies suggest that it can inhibit bone formation, while other indicate it may have no effect at all (Bourrin et al., 1994; Li et al., 1991; Morey & Bayklink, 1978; Ohashi et al., 2002; Robling et al., 2001; Sibonga et al., 2000; Steinberg & Trueta, 1981; Stokes et al., 2002; Turner, 1995).

A number of animal loading models have been developed, including rodent exercise studies, rodent whole body vibration, and in vivo loading models (Fritton et al., 2005; Iwamoto et al., 2005; Lee et al., 2002; Prisby et al., 2008; Turner et al., 1991; Wallace et al., 2007). An advantage of the in vivo mechanical loading models is that controlled, repeated mechanical forces are applied to the skeletal site of interest. In contrast, exercise studies are associated with mechanical stimuli that are much more difficult to quantify. One in vivo loading model is the mouse tibial axial loading model. This model is based on the application of cyclic loads to one tibia, using the contralateral unloaded tibia as a control (De Souza et al., 2005; Fritton et al., 2005).

We know very little about how mechanical forces that are transiently compressive (as opposed to sustain periods of compression) impact longitudinal bone growth (Foster, 2019; Villemure & Stokes, 2009).

Mechanical forces related to gravitational changes, ambulation, and exercise may contribute to modulate bone growth. Because daily physical activities transmit complex mechanical loads including tension, compression, torsion, and shear to the skeleton, simplified models such as axial loading provide an advantage in identifying the mechanisms by which bone growth may be affected by loading.

Our model applies cyclical, biologically relevant strains for a prolonged time period (4 weeks) to one tibia of juvenile mice, while using the contralateral one as an internal control.

We chose juvenile mice for our study due to the fact that, during the first 4–6 weeks of life, rodents experience the most rapid longitudinal bone growth. Thus, the model of juvenile rodent in studies on bone growth enables an investigator to identify even small, yet statistically significant, differences between experimental and control groups. The goal of our study was to analyze the effects of such loading on tibial longitudinal bone growth and identify the possible molecular mediators of these effects.

## 2 MATERIALS AND METHODS

### 2.1 Loading model

**FIGURE 1:**
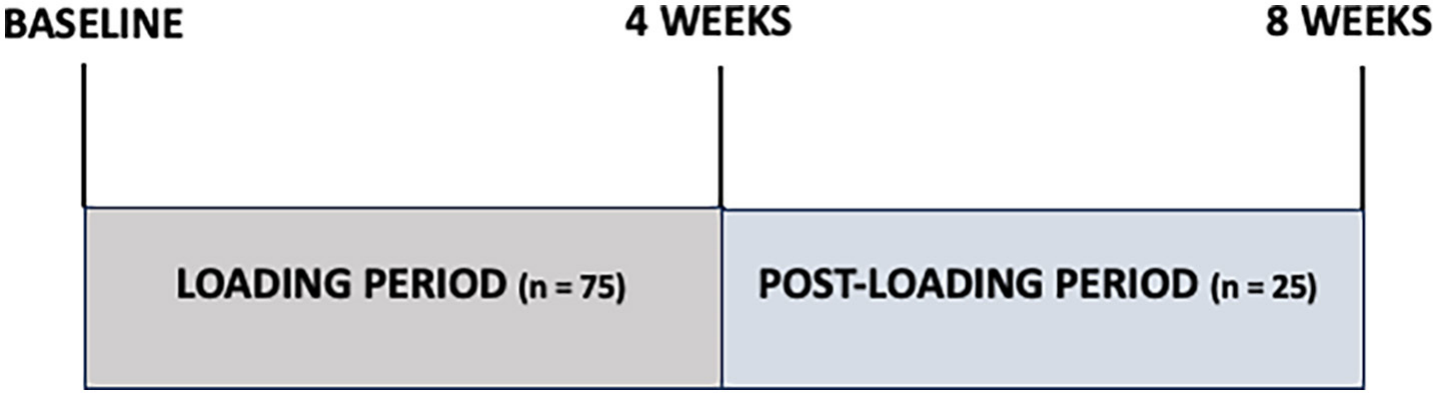
Schematic representation of the experimental study. Four‐week‐old TOPGAL mice (DasGupta & Fuchs, 1999) were exposed to 100 compressive loading cycles of 5 Newton (N) force to the right tibia using a Bose ElectroForce 3220 dynamic loading system. The left tibia was used as an unloaded control. X‐ray of the whole mouse were taken at baseline and at the end of the 4‐week loading period, tibial lengths measured and tibial growth calculated. At the end of the 4 weeks of loading period, mice were separated into two groups. One group was euthanized and mouse tibiae were isolated and processed for histology and RNA extraction. The other group was kept alive for four more weeks. Eight weeks after the beginning of the study, x‐ray were taken, tibial length measured, and tibial growth calculated for the whole 8‐week period in the previously loaded and unloaded tibiae.

Seventy‐five 4‐week old TOPGAL mice (DasGupta & Fuchs, 1999) were exposed to mechanical loading using a Bose ElectroForce 3220 dynamic loading system. Before each loading session, mice were anesthetized with 3.5% isoflurane. Each loading session included 100 compressive loading cycles of 5 Newton (N) force to the right tibia at the frequency of 2 Hz per cycle, as previously described (Lara‐Castillo et al., 2015) The left tibia was used as an unloaded control. Loading sessions were carried out three‐times a week, for 4 weeks. After 4 weeks of loading, mice were separated into two groups: one group (50 mice) was euthanized immediately after the last cycle, while the other group (25 mice) was kept alive (without further loading) and euthanized 4 weeks later (to assess eventual persistent effects of loading on longitudinal tibial growth 4 weeks after it had ceased) (Figure 1).

Whole body and tibial lengths were measured on x‐ray films taken at the beginning and the end of the 4‐week loading, and 4 weeks after the end of loading. Lengths of tibiae were measured using ImageJ (National Institutes of Health, MD) software.

Loaded and unloaded tibiae were isolated from mice euthanized at the end of the 4‐week loading period. Some tibiae were processed for microCT analysis. Growth plates were dissected from the remaining tibiae and used for RNA isolation or processed for histology.

Mice were purchased from the Jackson Laboratories and then housed and crossbred at the University of Maryland Kansas City Laboratory Animal Resource Center (LARC). Animals were housed in groups and fed with standard chow ad lib. Animal care was in accordance with the Guide for the Care and Use of Laboratory Animals (Department of Health, Education, and Welfare Publication [National Institutes of Health] 85–23, revised 1988). All procedures were approved by the Institutional Animal Care and Use Committee of the University of Missouri‐School of Medicine (Kansas City, Missouri).

Euthanasia was carried out by CO_2_ inhalation, followed by cervical dislocation.

### 2.2 Quantitative histology

**FIGURE 2:**
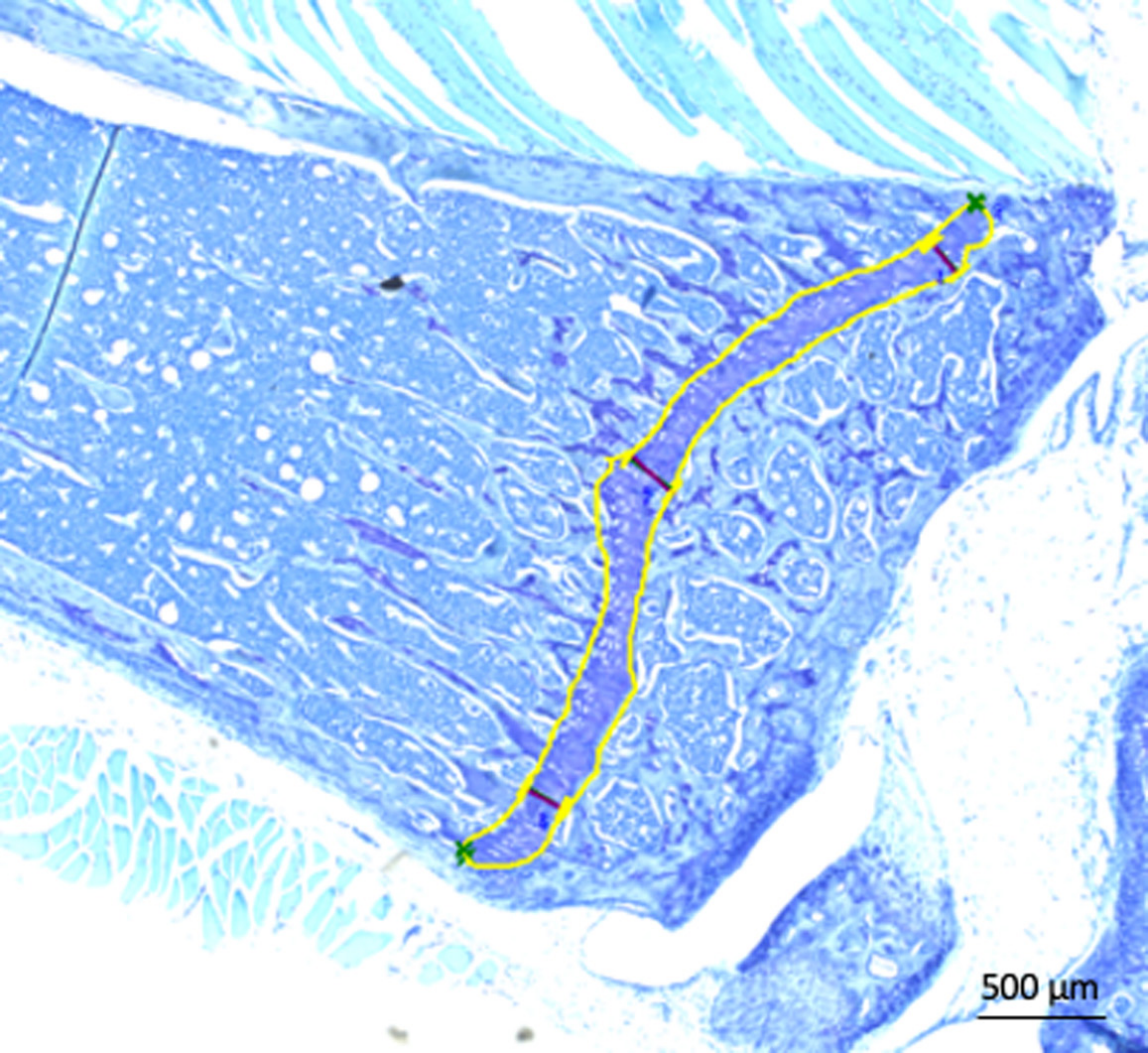
Representative photomicrograph of the mouse tibial growth plate. At the end of the 4‐week study period, mouse tibiae were isolated and, after routine histological processing, 5‐ to 7‐mm thick longitudinal sections from each bone were obtained and stained with toluidine blue. Growth plate images were captured using the Keyence BZ‐X800 microscope (Itasca, IL) and analyzed using Keyence BZ‐X800 software. Tibial growth plate height was measured at the center and at the two ends of the growth plate (vertical black bars), and the mean value calculated for each loaded and unloaded tibial growth plate. The area of the growth plate measured in μm^2^ by drawing a line along the perimeter of the whole growth plate (yellow line). All measurements were performed by a single observer blinded to the treatment regimen. The representative photo shown in the figure was obtained at 20× magnification.

At the end of the 4‐week study period, we harvested four to seven tibiae for each treatment group. Three 5‐ to 7‐mm‐thick longitudinal sections from each bone were obtained and stained with toluidine blue. Growth plate images were captured using the Keyence BZ‐X800 microscope (Itasca, IL) and analyzed using Keyence BZ‐X800 software. Height of the epiphyseal, proliferative, and hypertrophic zone, and of the whole growth plate was measured at the center and at the two ends of the growth plate (Figure 2), and the mean value calculated for each loaded and unloaded tibial growth plate. The area of the growth plate was measured by drawing a line along the perimeter of the whole growth plate (Figure 2). All measurements were performed by a single observer blinded to the treatment regimen. The representative picture shown in the figure was obtained at 20× magnification.

### 2.3 RNA extraction and real‐time PCR

Tibial growth plates to be used for RNA extraction were isolated, placed in tubes of TRIzol Reagent (ThermoFisher, Whaltam, MA, USA) and then in a −80°C freezer. Once thawed, the growth plates and TRIzol reagent were placed in 1.5‐mL Navy Eppendorf RNA lysis tubes (Next Advance, Troy NY, USA) and pulverized in a Bullet Blender (Next Advance). Once homogenized, the solution was placed in a new Eppendorf tube, and 0.2 mL of chloroform (ThermoFisher) per 1 mL of TRIzol reagent was added. Tubes were shaken vigorously for 15 s and then incubated at room temperature for 3 min. Following incubation, tubes were spun at 11,600 × *g* for 15 min at 4°C. Following centrifugation, the upper aqueous phase was collected and placed in a new Eppendorf. The QIAGEN RNeasy minikit (QIAGEN Inc, Germany. Cat. No/ID 74104) was used to isolate the RNA. The isolated RNA was stored at −80°C. The recovered RNA was further processed using iScript™ cDNA Synthesis Kit (Bio‐RAD, CA. Cat. No. 1708891) to produce cDNA. The cDNA products were directly used for PCR or stored at −20°C for later analysis. Real‐time quantitative PCR was carried out using the Applied Biosystems ViiA 7 real‐time PCR system (ThermoFisher) with a final volume of 10 μL containing 250 ng cDNA, 5 μL iTaq Universal SYBR Green Supermix (Bio‐RAD. Cat. No. 1725121), 1 μM primers (IDT, IA) in 3.5 μL of nuclease‐free water. The conditions for RT‐qPCR reactions were one cycle at 95°C for 15 s followed by 40 cycles at 95°C for 3 s and annealing at 60°C for 30 s. Results were normalized to the housekeeping gene 18 s ribosomal RNA (18 s). Relative gene expression from different groups was calculated with the 2ΔΔCT method and compared with the expression level of appropriate control samples. Specific primer sequences for individual genes are listed below.

**Table:** 

Genes	Forward	Reverse
18s	TAAAGGAATTGACGGAAGGGCA	ATCTGTCAATCCTGTCCGTGTC
Col2a1	AGCAGCAAGAGCAAGGAA	TGGACAGTAGACGGAGGAA
Col10a1	ATAAGAACGGCACGCCTACGATGT	CTGCATTGGGCAATTGGAGCCATA
Igf1	GGGCATTGTGGATGAGTGTTGCTT	TGGAACGAGCTGACTTTGTAGGCT
Pthrp	CATCAGCTACTGCATGACAAGG	CTGTGTGGATCTCCGCGAT
Ctnnb1	ATGGAGCCGGACAGAAAAGC	TGGGAGGTGTCAACATCTTCTT

### 2.4 microCT

microCT was performed using a Scanco vivaCT40 system. The resolution of the scans was set at 10.5μM. Trabecular bone data were collected from a 1‐mm region (100 slices) of the proximal tibia, distal to the growth plate, and cortical bone data collected from a 0.5‐mm region (50 slices) at midshaft. 3D and 2D morphometric parameters were calculated for trabecular and cortical bone selected regions of interest using CT Analysis Software. Surface rendering 3D models of trabecular and cortical bone were constructed using CT Vox software.

Three‐dimensional analysis was performed to determine the percentage of total bone volume relative to the total area of interest (BV/TV) and trabecular bone density (MMD).

## 3 RESULTS

All the individual datapoints relative to our experimental work are available in the Supplemental File—Data S1.

### 3.1 Effects of intermittent weight loading on mouse tibial longitudinal growth

**FIGURE 3:**
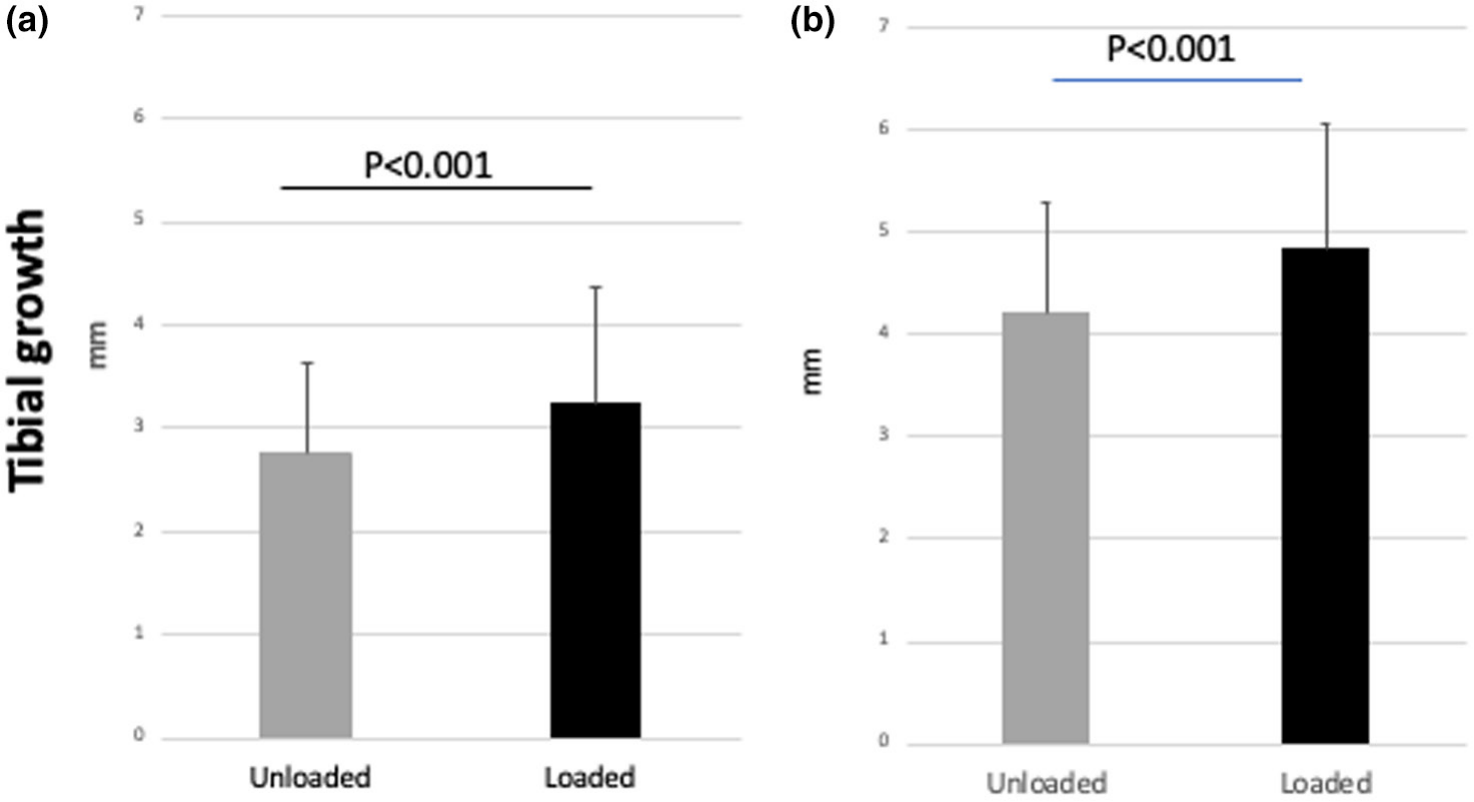
Effects of intermittent weight loading on tibial longitudinal growth. The right tibiae of 4‐week‐old mice (*n* = 75) were exposed to 100 compressive loading cycles of 5 N force using a Bose ElectroForce 3220 dynamic loading system. Such cycles were applied 3 times a week for 4 weeks. The left tibiae were used as unloaded controls. Tibial lengths were measured on x‐ray films taken at the beginning and the end of the 4‐week loading, and tibial growth of loaded and unloaded tibia calculated in the whole sample (difference between lengths measured at the 4‐week timepoint and at baseline) ((a), *n* = 75). At the end of the 4‐week loading period, mice were separated into two groups: One group was euthanized, while the other group remained in the study for further analysis. Four weeks later (4 weeks after the end of the loading period and 8 weeks from the beginning of the study), x‐ray films were taken, tibial lengths measured, and mean tibial growth calculated (difference between lengths measured at the 8‐week timepoint and at baseline) ((b), *n* = 25) (mean ± SE).

**FIGURE 4:**
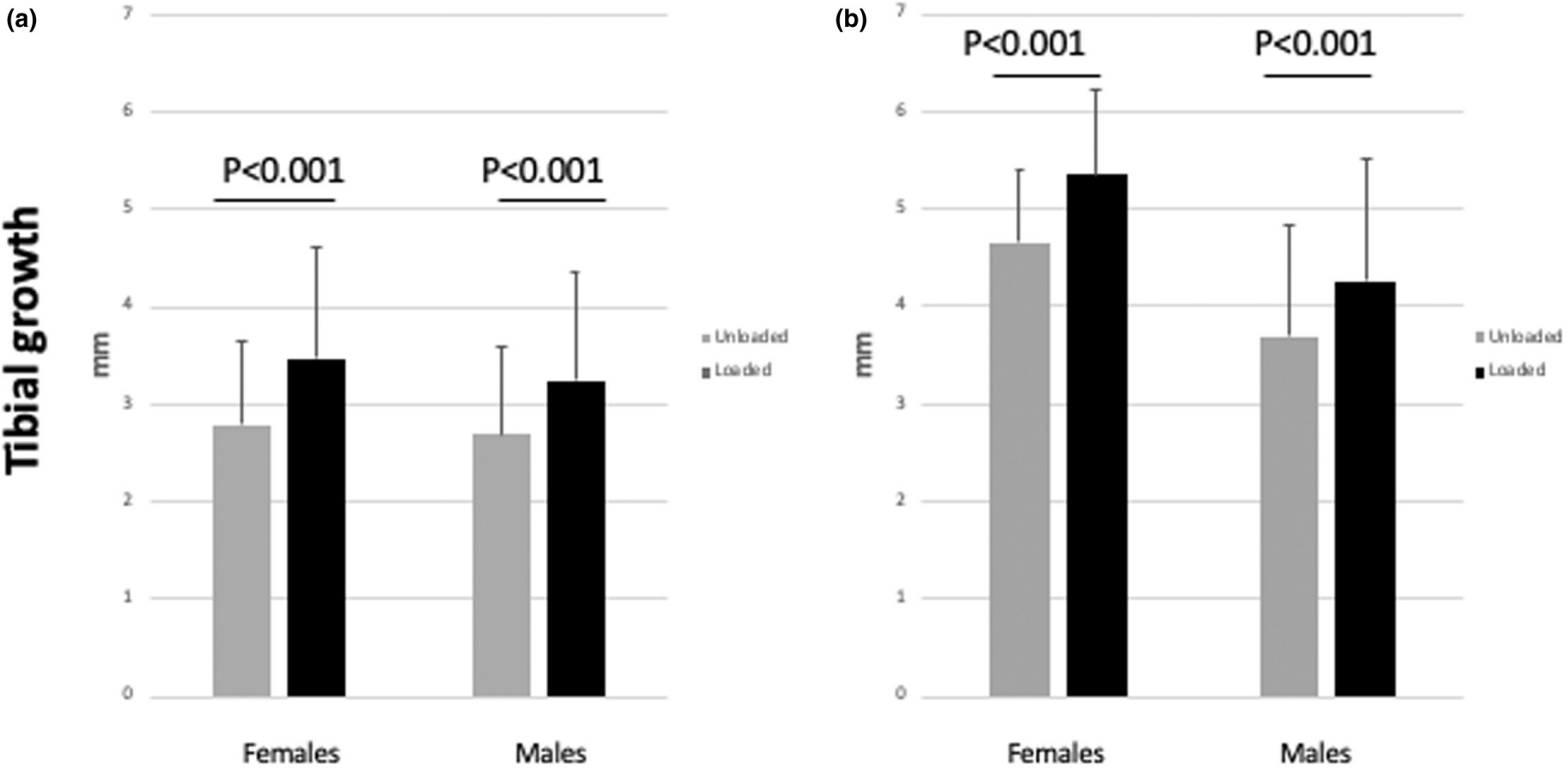
Effects of intermittent weight loading on tibial longitudinal growth of male and female mice. Tibial lengths were measured on x‐ray films taken at the beginning and the end of the 4‐week loading period, and tibial growth calculated separately in the male and female mouse subgroups ((a) males, *n* = 32; females, *n* = 43). At the end of the 4‐week loading period, mice were separated into two groups: One group was euthanized, while the other group remained in the study for further analysis. Four weeks later (4 weeks after the end of the loading period and 8 weeks from the beginning of the study), x‐ray films were taken, tibial lengths measured, and mean tibial growth calculated separately in male and female mice ((b) males, *n* = 12; females, *n* = 13) (mean ± SE).

At the beginning of the 4‐week loading period, the mean length of the loaded and unloaded tibiae was equivalent (supplemental file—Data S1). By the end of the 4‐week loading period, both unloaded and loaded tibiae experienced longitudinal growth. However, the mean tibial growth of the loaded tibiae was significantly greater than that of the unloaded tibiae, in the whole sample of mice (Figure 3a, *n* = 75, 32 male and 43 female mice) as well as when the effect of loading on longitudinal growth was studied separately in male and female mice (Figure 4a). While 50 of the 75 mice were euthanized at the end of the 4‐week loading period, the remaining 25 mice (12 male and 13 female mice) were kept alive and evaluated 4 weeks after the end of the loading period (8 weeks after the beginning of the study). At that timepoint, the mean tibial growth of the previously loaded tibiae remained greater than that of the unloaded ones, both in the whole sample of 25 mice (Figure 3b) and in the male and female mouse subgroups (Figure 4b). No difference was found between the mean tibial growth of males and females (supplemental file—Data S1).

### 3.2 Effects of intermittent weight loading on mouse tibial growth plate height and area

**FIGURE 5:**
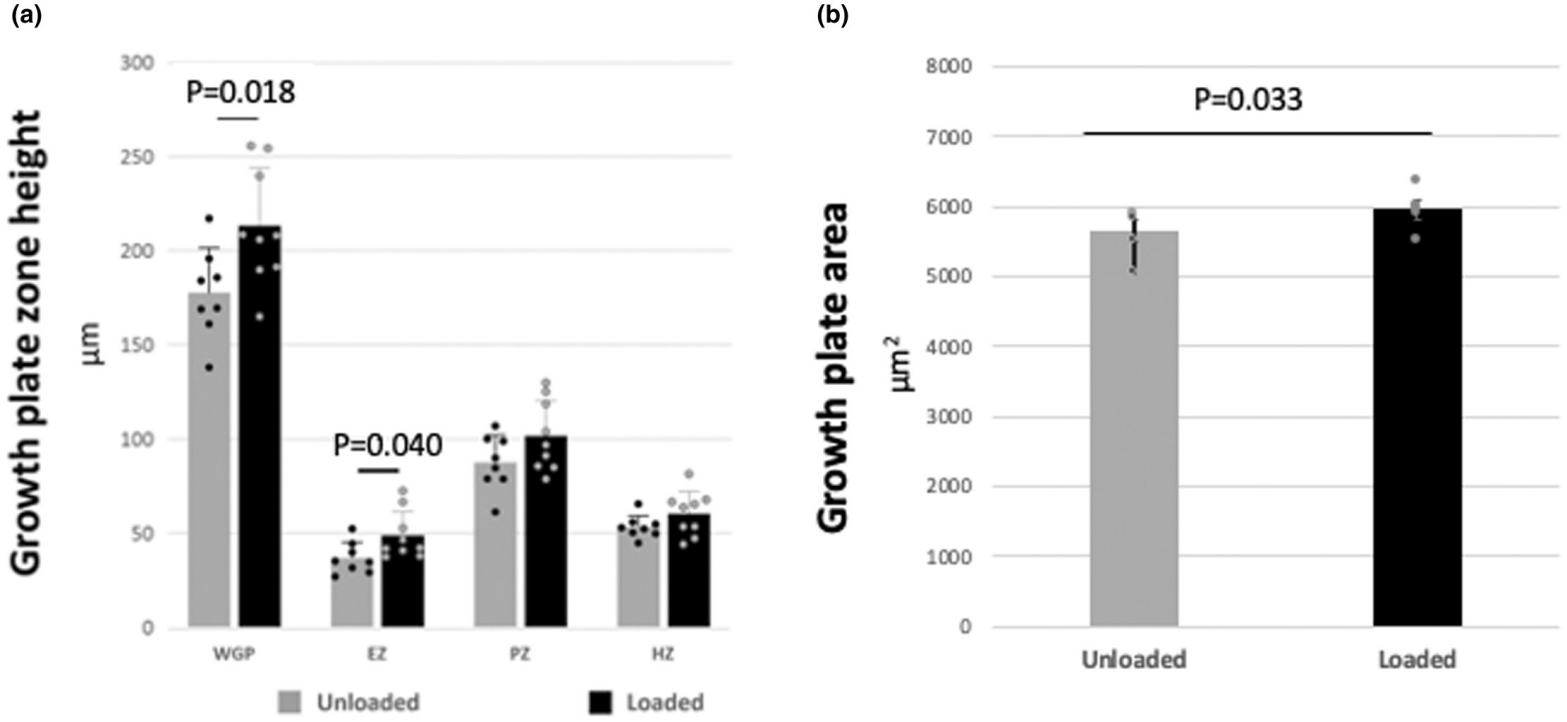
Effects of intermittent weight loading on mouse tibial growth plate height and area. At the end of the 4‐week loading period, loaded and unloaded were isolated and three 5‐ to 7‐mm‐thick longitudinal sections from each bone were obtained and stained with toluidine blue. Growth plate images were captured using the Keyence BZ‐X800 microscope (Itasca, IL) and analyzed using Keyence BZ‐X800 software. Tibial growth plate epiphyseal, proliferative and hypertrophic zone, and of the whole growth plate, heights were measured in μm at the center and at the two ends of the growth plate, and the mean value calculated for each loaded and unloaded tibial growth plate ((a), *n* = 9). The area of the growth plate was measured in μm^2^ by drawing a line along the perimeter of the whole growth plate ((b), *n* = 5). All measurements were performed by a single observer blinded to the treatment regimen. The mean heights of the growth plate epiphyseal zone and of the whole growth plate of the loaded tibiae were significantly greater than those of the unloaded tibiae. The mean area of the growth plate of the loaded tibiae was greater than that of the unloaded ones (mean ± SE).

At the end of the 4‐week loading period, the mean heights of the epiphyseal zone and of the whole growth plate of the loaded tibiae were significantly greater than those of the unloaded tibiae (Figure 5a, *n* = 8–9). The mean growth plate area of the loaded tibiae was greater than that of the unloaded ones (Figure 5b, *n* = 5).

### 3.3 Effects of intermittent weight loading on the expression of growth factors in the mouse tibial growth plates

**FIGURE 6:**
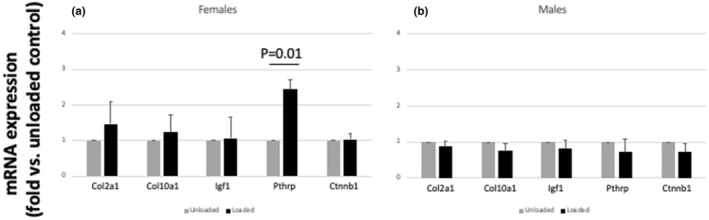
Effects of intermittent mechanical loading on the expression of Ctnnb1, Igf1, and Pthrp in the growth plate of loaded and unloaded mouse tibiae. At the end of the 4‐week loading period, total RNA was extracted from the tibial growth plate of the loaded and unloaded tibia (= 6 mice). mRNA expression was quantitated by real‐time qPCR. Results were normalized to the housekeeping gene 18s ribosomal RNA (18s). Relative gene expression from different groups was calculated with the 2ΔΔCT method and compared with the expression level of appropriate control samples. Results were expressed as fold change compared to unloaded bones, in female (a) and male (b) mice (mean ± SE).

We then studied the mRNA expression of some of the best known growth‐promoting growth factors in the growth plate by qPCR (IGF‐1, PTHrP, beta‐catenin). At the end of the 4‐week loading period, we found a greater expression of PTHrP in the loaded tibial growth plates only in female mice (Figure 6a). No difference in the expression of IGF‐1 and beta‐catenin was found between loaded and unloaded tibial growth plates, neither in female nor in male mice.

### 3.4 Effects of intermittent weight loading on tibial bone structure

We analyzed the effects of intermittent loading on bone architecture by microCT. With respect to all parameters analyzed (cortical bone fraction, trabecular bone fraction, trabecular number, trabecular separation, and trabecular bone density) we found no differences between loaded and unloaded tibiae, nor between male and female mice (11 mice—6 female and 5 male) (Table 1).

**TABLE 1:** Effects on intermittent loading on tibial bone architecture analyzed by microCT.

Microct parameters				
	Loaded		Unloaded	
	Males	Females	Males	Females
C BV/TV (%)	99.58 ± 0.02	99.44 ± 0.04	99.52 ± 0.01	99.59 ± 0.01
TB BV/TV (%)	12,36 ± 0.05	12.89 ± 0.03	11.59 ± 0.03	13.39 ± 004
TB MD (mm HA/cm^3^)	911.60 ± 14.54	906.73 ± 33.44	912.01 ± 15.39	927.96 ± 15.28
TB number (1/mm)	5.86 ± 2.02	4.97 ± 1.48	5.92 ± 1.27	4.78 ± 1.17
TB separation (mm)	0.18 ± 0.07	0.21 ± 0.06	0.17 ± 0.03	0.22 ± 0.05

*Note*: Mean ± SD.

Abbreviations: BV, bone volume; C, cortical; MD, mineral density; TB, trabecular; TV, total volume.

## 4 DISCUSSION

Longitudinal bone growth results from a process called endochondral ossification. In this process, chondrocytes proliferate and hypertrophy in the epiphyseal growth plate. The new cartilage formed via chondrocyte proliferation and hypertrophy is replaced by bone tissue. The cellular events responsible for endochondral ossification and longitudinal bone growth are regulated by local and systemic growth factors, which in turn are regulated by genetic factors and environmental ones, such as nutrition, gravity, and physical activity.

Gravity and physical activity generate mechanical forces on the long bones and on the growth plates that may be involved in the regulation of bone growth.

However, these forces result from a combination of various strain types (tensions compression, shear), and it is difficult to dissect the effects of these individual strains. Experimental studies have confirmed these challenges: some studies have shown an increase in bone length from treadmill and jumping exercises (Hammond et al., 2010; Umemura et al., 1995), while others have described growth suppression (Bourrin et al., 1994; Li et al., 1991). These conflicting findings may depend on variations in magnitude, frequency, type of loads, and on whether the mechanical forces are transient (intermittent) or sustained. To bypass these limitations, we have used an experimental method that is based on the consistency of the mechanical loading applied to bone.

To mimic the effects of mechanical forces exerted during human ambulation, we chose a model of cyclic (intermittent) compression on the mouse tibia at a force of 5 N, which is a strain magnitude similar to those measured during normal ambulation (Mosley et al., 1997).

After 4 weeks of cyclic loading, the growth of the mouse loaded tibiae was significantly greater than those of the contralateral unloaded tibiae. Of note, the stimulatory effect of mechanical loading on tibial growth persisted in the 4 weeks following the cessation of loading.

In addition, at the end of the 4‐week loading period the whole growth plate and the epiphyseal zone heights, as well as the overall area of the loaded tibial growth plates were significantly greater than those of the unloaded growth plates. Such finding suggests a loading‐mediated enhanced growth plate chondrocyte formation.

In the study of Ohashi et al. (2002), cyclic compressive forces of three different magnitudes (17, 8.5, and 4 N) were applied to growing rats' ulnas for 10 min a day for 8 days. At the end of the experiment, the length of the ulnae loaded with 17, 8.5, and 4 N force magnitude was shorter than that of the unloaded ones. However, in the group loaded with 4 N magnitude the difference in length was only 0.8%. Furthermore, the authors measured the final length of the ulnae after 8 days of loading rather than the true ulnar growth (in these groups, the baseline length was not provided).

A similar study was carried out by Robling et al. (2001) on rat ulnae loaded for 2 weeks with 17 or 8.5 N force magnitudes. At the end of the loading period, the length of the ulnae loaded with either 17 or 8.5 N forces was shorter than the unloaded contralateral bones (with the difference being ~0.7% in the group loaded with 8.5 N force). In the ulnae loaded with 17 N force, the reduction in length was twice that exhibited by the loaded ulnae with 8.5 N. The growth plates of the ulnae loaded with 17 N were thicker than the contralateral unloaded growth plates, while no difference in thickness was found in the ulnae loaded with 8.5 N. Of note, both of these force magnitudes were greater than the one used in our study. Consistent with our findings, Zhang P and others (Zhang et al., 2010) have shown that forces of 0.5 N applied to the hindlimbs of 8‐week old mice induced an increased length of the loaded femur and tibia, compared to unloaded contralateral bones. The thickness of the loaded growth plates was increased as well.

Thus, our findings regarding longitudinal bone growth at the end of the loading period and previous similar studies in rodents indicate that elevated loading forces tend to inhibit longitudinal growth, while strains of lower intensity (like ours) tend to stimulate it. Differences between our findings and others may be related to the duration of the loading period, the species (mouse vs. rat) and the type of bone (tibia vs. ulna) used.

β‐Catenin and IGF‐1 are two critical growth factors in growth plate function and endochondral ossification. Deletion of β‐catenin causes growth plate abnormality with delayed cartilage calcification (Akiyama et al., 2004). The postnatal conditional knockout (KO) of β‐catenin induces reduction in collagen 10‐positive hypertrophic chondrocytes and abnormal cell alignment with loss of slow‐cycling cells in the resting zone of the growth plate (Dao et al., 2012). Deficiency of β‐catenin in Col10a1 expressing cells impairs late terminal differentiation of chondrocytes and strongly reduces subchondral trabecular bone formation (Golovchenko et al., 2013). Thus, Wnt/β‐catenin signaling regulates multiple steps of chondrocyte function in the growth plate, including proliferation, matrix production, hypertrophy and replacement to bone in direct and indirect manners (Akiyama et al., 2004; Dao et al., 2012).

IGF‐1 is a critical modulator of longitudinal bone growth and endochondral ossification, acting both as an endocrine factor and a paracrine factor expressed in the growth plate (Dixit et al., 2021).

In our study, we found no change in expression of β‐catenin and IGF‐1 in the loaded tibial growth plates when compared to the unloaded ones. Previous evidence indicates that mechanical strain modulates the expression of β‐catenin/Wnt genes and IGF‐1 in osteoblasts and osteocytes (Hens et al., 2007; Rejinders et al., 2007; Robinson et al., 2006). On the other hand, no prior study has analyzed the expression of β‐catenin in long bones' growth plate chondrocytes exposed to mechanical loading. In one study, a single period of mechanical loading on the rat tibia did not induce any change in the expression of IGF‐1 in chondrocytes (Rejinders et al., 2007).

After 4 weeks of loading, we found a greater Pthrp gene expression in the growth plate chondrocytes of the female mouse loaded tibiae, while no difference was found in male mice. This finding suggests that mechanical loading may have a sexually dimorphic effect on Pthrp expression and suggests, at least in female mice, that the increased expression of Pthrp may be responsible of the increased tibial longitudinal growth and growth plate cartilage formation induced by cyclic, compressive forces on long bones.

PTHrP and its receptor (PTHR1) are expressed in the growth plate, and they both exert a significant regulatory role in growth plate development and function; PTHrP appears especially necessary for the normal proliferation of growth plate chondrocytes (Martin et al., 2021).

Xu et al. have previously published a study on the regulation of PTHrP expression by cyclic mechanical strain (Xu et al., 2013). In this study, they demonstrated an increased PTHrP expression in cultured rat growth plate chondrocytes subjected to cyclic tensile strain of varying magnitude and duration.

Lastly, 4‐weeks of cyclic mechanical loading at 5 N did not result in any significant change of trabecular or cortical bone mass, neither in male nor in female mice. In a previous knee loading study carried out using a mechanical strain at 0.5 N (Zhang et al., 2010), the authors described a modest, although statistically significantly increased tibial bone mineral density and bone mineral content in the loaded tibiae. However, these parameters were analyzed 2 weeks after the completion of 10‐day loading period.

Unlike our study, a number of prior studies in rodents have reported a significant enhancement of osteogenic response and cortical and trabecular bone mass induced by tibial loading (Main et al., 2014; Warden et al., 2014; Weatherholt et al., 2012; Yang et al., 2017, 2021). In our opinion, the explanation for these discordant findings lies in the multiple differences between our experimental model and those used by other authors. Compared to most of these prior models, the mice studied by us were significantly younger. Furthermore, the total loading time in our study was longer (4 weeks, while most of the other studies used from single loading sessions up to 2 weeks of loading). Lastly, we used a lower number of cycles/events per loading session and a smaller strain magnitude compared to the studies which described a loading‐mediated increased cortical and trabecular bone mass.

We acknowledge that the variability in sample size regarding the several parameters analyzed is an important limitation of our study. While the number of mice studied to determine the effects of loading on tibial growth was very high, we were able to process only a few tibiae for histology and bone architecture, due to significant technical difficulties.

In addition, the difference in the number of data points regarding the measurements of the growth plate height and the growth plate area depended on the fact that some bone sections did not include the whole growth plate. Thus, the growth plate area could not be measured in all the bone sections.

In conclusion, our findings suggest that intermittent compressive forces applied on tibiae at mild–moderate strain magnitude (similar to those occurring during physiological conditions such as normal ambulation) induce a significant and persistent longitudinal bone growth. PTHrP expressed in the growth plate appears to be one growth factor responsible for stimulating endochondral ossification and bone growth in female mice after mechanical loading. Further studies are needed to better identify additional molecular mechanisms underlying this mechanical loading‐dependent bone growth acceleration.

## FUNDING INFORMATION

Our experimental work has been supported by institutional internal funding.

## ETHICS STATEMENT

This manuscript has not been previously published elsewhere. All the authors meet the criteria for authorship and have no conflict of interest to declare.

## Supporting information


Data S1.

## Data Availability

The data that supports the findings of this study are available in the supplementary material of this article.
